# Regulation of rhythm genesis by volume-limited, astroglia-like signals in neural networks

**DOI:** 10.1098/rstb.2013.0614

**Published:** 2014-10-19

**Authors:** Leonid P. Savtchenko, Dmitri A. Rusakov

**Affiliations:** UCL Institute of Neurology, University College London, Queen Square, London WC1N 3BG, UK

**Keywords:** neural network, basket cell, astrocyte

## Abstract

Rhythmic activity of the brain often depends on synchronized spiking of interneuronal networks interacting with principal neurons. The quest for physiological mechanisms regulating network synchronization has therefore been firmly focused on synaptic circuits. However, it has recently emerged that synaptic efficacy could be influenced by astrocytes that release signalling molecules into their macroscopic vicinity. To understand how this volume-limited synaptic regulation can affect oscillations in neural populations, here we explore an established artificial neural network mimicking hippocampal basket cells receiving inputs from pyramidal cells. We find that network oscillation frequencies and average cell firing rates are resilient to changes in excitatory input even when such changes occur in a significant proportion of participating interneurons, be they randomly distributed or clustered in space. The astroglia-like, volume-limited regulation of excitatory synaptic input appears to better preserve network synchronization (compared with a similar action evenly spread across the network) while leading to a structural segmentation of the network into cell subgroups with distinct firing patterns. These observations provide us with some previously unknown insights into the basic principles of neural network control by astroglia.

## 1. Introduction

Synchronized oscillations in neural circuits could provide, at least theoretically, a universal device for pattern recognition and information handling in the brain [1,2]. Such periodic activities are in many cases driven by self-sustained rhythms generated by interconnected populations of inhibitory gamma-aminobutyric acid (GABA)ergic interneurons, which receive multiple excitatory inputs from principal cells [3–5]. In the hippocampus, rhythm generation in interneuronal networks is thought to depend on the combined action of incoming excitatory synaptic currents and of the slower changes in the concentration of extracellular (ambient) GABA [4,6,7]. The astroglia-controlled GABA uptake and recycling machinery [8] thus provides one plausible mechanism to control oscillatory activities in local neural populations. It has also emerged that astrocytes can respond to physiological stimuli by releasing a variety of signalling molecules that target local synaptic receptors (reviewed in [9–12]). Among such molecules feature ATP (which, once released into in the extracellular space, is degraded to adenosine) [13,14] and an *N*-methyl-d-aspartate (NMDA) receptor co-agonist d-serine [15,16]. Adenosine release from astroglia has been associated with activation of presynaptic purinergic A1 receptors [17,18] (however [19]), the action which in most cases lowers (in some cases by more than 50%) release probability at excitatory connections, thus moderating signal transfer through local synaptic networks [20,21]. At the same time, Ca^2+^ waves in astrocytes have also been associated with increased release probability at nearby synapses [22], which could involve a presynaptic action of astroglia-released glutamate [23] and manifest itself as a transiently elevated (sometimes by several-fold) frequency of spontaneous synaptic discharges [23,24]. Furthermore, astroglial release of d-serine can boost the availability of high-affinity, predominantly postsynaptic NMDA receptors, thus enabling potentiation of excitatory transmission [25–27].

Astroglia therefore should be capable, at least in certain conditions, of triggering substantial variations in the release probability at local synaptic connections. In turn, changes in the intensity (amplitude or frequency) of the excitatory drive have long been known to play an important regulatory role in neural network oscillations [5,28–31]. While synaptic connections to or from individual neurons can spread over hundreds of micrometres, mixing in space with similar connections to or from thousands of other neurons, individual astrocytes occupy separate, non-overlapping tissue domains [32]. Thus, they might, in principle, exert a regulatory action within a volume-limited population of excitatory, and perhaps inhibitory, synapses [26,33] in the local neuronal network. Whether such volume-limited synaptic regulation produces network effects that differ qualitatively from the effects of a spatially homogeneous synaptic efficacy change is not known. To address this question, and thus to understand the potential adaptive role of astrocyte-like network regulation, here we explored a well-tested theoretical network of hippocampal interneurons (basket cells) which displays physiologically plausible oscillatory behaviours [7,34–36].

## 2. Material and methods

### (a) Basket cell network with a spatial neighbourhood factor

**Figure 1.**
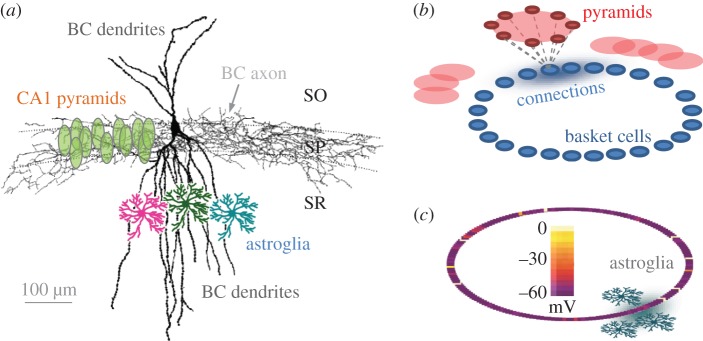
Simulated network of basket cells–pyramidal cells. (*a*) A diagram illustrating characteristic morphology of a reconstructed basket cell (BC, modified from [37]), the extent of its dendritic and axonal trees in hippocampal area CA1, and relative arrangements of pyramidal cell bodies and astrocytes (showing several examples only), as indicated. SO, SP, SR: strata oriens, pyramidale, radiatum*,* respectively. (*b*) A schematic illustrating the architecture of the simulated BC network (not to scale), with each BC (blue) receiving excitatory synaptic input from a pyramidal cell subnetwork (red); blue shadow (connections) qualitatively depicts the Gaussian distribution of cell–cell connection weights centred at a given BC. (*c*) A simulation snapshot of membrane voltage for the 200-cell BC network (as depicted in *b*); scale bar, voltages in pseudo-colours. Grey shadow illustrates the spread of a volume-limited (astroglia-like) effect on synaptic transmission mimicking the local action of astrocytes. See §2 Material and methods and figures for further detail.

The neural network design was generally based on the well-tested hippocampal basket cell (BC) network incorporating excitatory inputs from principal neurons (pyramidal cells; figure 1*a* depicts the characteristic BC arrangement in hippocampal area CA1) [34]. This cell population has classically been associated with experimentally documented high-frequency oscillations of local field potential (and the corresponding synchronizations of cell spiking) that appear to be related causally to certain behaviour traits or memory-forming tasks [31,38–40]. The network model was built using the NEURON computational environment [41] and included 200 fast-spiking interneurons (BCs) interconnected via typical inhibitory synapses (GABA potential = −65 mV). The waveforms of GABAergic synaptic currents were modelled using the dual-exponential formalism incorporated in the *Exp2Syn* function of the NEURON simulator: synaptic conductance followed the time course 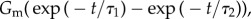 where *G*_m_ is the maximal conductance and *τ*_1_ = 2.5 ms and *τ*_2_ = 10 ms are the rise and the decay time constants, respectively.

For computational design purposes, connections among BCs were represented by a virtual ring: this common configuration allows an exhaustive representation of cell–cell links while avoiding issues pertinent to boundary conditions [7,34–36]. Note that the ring represents the matrix of cell–cell connections rather than the actual three-dimensional geometry of the BC network. However, cell positions in the ring do reflect their neighbourhood relationships: for example, adjacent neighbours in the ring generally correspond to spatial neighbours in the real network. To reflect non-instantaneous signal propagation between adjacent cells, an approximately 50 μm virtual spacing was introduced computationally between nearest neighbours, as detailed earlier [42]. To account further for some previously established parameters of the BC network organization, the network incorporated an additional neighbourhood factor: each cell was randomly connected to a subpopulation of its 100 nearest neighbours by inhibitory synapses with a connection probability of 0.57, reflecting anatomical analyses of functional links among parvalbumin-positive interneurons in area CA1 [43] (example in figure 1*a*). The signal effective propagation radius for the entire network was approximately 2 mm, which was comparable to the characteristic dimensions (hence spike-release latencies) of the hippocampal CA3 or CA1 network in the murine.

### (b) Synaptic input from pyramidal cells

The classical BC network models use steady-state depolarization as the form of excitatory input from principal cells to individual interneurons [31,34]. To improve physiological compatibility of the model, the present simulated network has incorporated realistic barrages of excitatory postsynaptic currents (EPSCs; NEURON *Exp2Syn* function: *τ*_1_ = 0.5 ms rise time and *τ*_2_ = 5 ms decay time, respectively) generated in individual BCs by synaptic inputs from simulated networks of pyramidal cells (figure 1*b*), as described recently [36]. When required, this arrangement also allows tonic (quasi-steady-state) excitation using evenly distributed low-frequency synaptic input. The synaptic input parameters were adjusted as follows. Because BCs normally host approximately 2000 synaptic inputs from pyramidal neurons and because pyramidal neurons fire at an average frequency of 1–2 Hz [34,35,37,40], the combined occurrence of incoming spikes should be in the range 2–4 kHz. Given the release probability of 0.2–0.3, this corresponds to an expected barrage of EPSCs at a frequency of 400–1000 Hz assuming 100% active connections. Indeed, in our simulations, we have found that EPSCs generated at individual interneurons at, on average, approximately 300 Hz lead to the network oscillation and synchronization behaviours that were fully compatible with those under ‘baseline’ tonic current conditions used in the classical BC network models [31,34].

### (c) Computational elements

The network was simulated using the NEURON computational environment, as described in detail previously [36,44,45] (the generic model can be downloaded at https://senselab.med.yale.edu; ModelDB accession number 138421), based on a well-established generic network design [34,46]. In brief, the model interneuron had a cylindrical shape (length 62 μm; diameter 62 μm; axial resistance 100 ohm cm^−1^; Cm 1 µF cm^−2^; resting membrane potential −65 mV). Cell membrane properties were described using Hodgkin–Huxley formalism (action potential threshold approx. −58 mV, determined from the voltage response to a ramp of excitatory current of 1 pA ms^−1^). The kinetics of the channels were typical of CA3 hippocampal fast-spiking basket cells [34]; the cell model was obtained from ModelDB (https://senselab.med.yale.edu; accession number 21329). Excitatory synaptic inputs to individual BCs were supplied by a network of 2000 interconnected pyramidal cells generating Poisson's series of spikes, with the average release probability ranging from 0.0 to 0.45 (figure 1*b*), which was enabled by the NetStim routines of NEURON. Thus, the output of pyramidal cell activity was represented by a stochastic glutamatergic synaptic currents (*V*_rest_ = 0 mV, see §2b for the kinetics) generated at individual BCs with an adjustable frequency *f* and postsynaptic conductance amplitude *g*. In baseline conditions, the stochastic excitatory synaptic input to individual interneurons was set at the average synaptic discharge frequency of *f*_0_ = 300 Hz, in line with earlier estimates, giving a sustained network oscillation frequency of 18–22 Hz (close to the gamma-frequency range for CA1 or CA3 interneuronal networks). Computations were carried using an in-house 64-node PC cluster optimized for parallel computing [47] (algorithms provided by Sitrus LLC, Boston).

### (d) Network monitoring parameters

The average network oscillation frequency *Ω* was determined as the greatest spectrum harmonic in the time series represented by the probability density (frequency histogram) of all spikes occurring in the network over time. Mean cell firing frequency *φ* was computed as the average number of all BC discharges over a given time period. Thus, when increases in the number if non-firing (silent) BCs in the network occur without changes in *Ω* the value of *φ* increases accordingly, and vice versa.

Network synchronization was evaluated as the mean coefficient of synchronization *k*(*τ*) representing an average value among coefficients *k_ij_*(*τ*) calculated for each (*i,j*) neuronal pair in the network over a 1000 ms interval, as follows (also see [36,45]). First, the 1000 ms interval was divided into *N* equal bins, each lasting *τ* = 0.1/*Ω*. For every selected pair of neurons *X* and *Y*, the spike occurrence within each *m*th binned time interval was represented by binary functions *X*(*m*) and *Y*(*m*) taking a value of zero or unity depending on whether the corresponding neuron generated one or no spikes, respectively (the occurrence of more than one spike within the binned time interval representing 1/10 of the average spiking period was negligibly small). Next, for each (*i,j*) pair of neurons, *k_ij_*(*τ*) was calculated using the formula
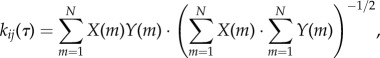
which gives a weighted measure of temporal spike coincidence for neuronal pairs over the sampled interval of 1000 ms. Finally, the mean coefficient of synchronization *k*(*τ*) was calculated as the global average of *k_ij_*(*τ*) for all neuronal pairs.

## 3. Results

### (a) Exploring space-constrained, astroglia-like influence in a neural network

Characteristic dimensions of hippocampal BCs and local astroglia (figure 1*a*) suggest that the release of signalling molecules (gliotransmitters) from an individual astrocyte might affect 5–10% synapses on each local BC. In addition, in contrast to pyramidal cells and many other neuronal types, BCs are distributed in the hippocampus without any significant overlap between their dendritic trees (figure 1*a*) [31,38–40]. Therefore, it appears plausible that astroglial influence could, at least in some conditions, affect only one or a small proportion of BCs in their entire network.

As discussed in §1, astroglial release of glutamate could reportedly lead to a substantial (up to several-fold) increase in release probability at nearby synapses [22–24], whereas release of adenosine from astrocytes could have a diametrically opposite effect [20,21]. To mimic such physiological actions in the modelled network, we explored variable changes in the discharge frequency *f* of excitatory synaptic inputs to one or more networked BCs. In the present context, our principal aim was to understand the implications of the fact that individual astroglia occupy non-overlapping, volume-limited tissue domains [32]. We therefore applied the synapse-modifying action to variable-size clusters of neighbouring BCs and compared the outcome with that under a quantitatively identical action distributed among randomly selected BCs. The comparison between these two scenarios was thus used to ask whether astroglia-like, volume-limited influences differ qualitatively from similar influences distributed evenly randomly, or as a ‘tonic’ effect, within the BC network. For the sake of clarity, and because the BC network configuration and its basic behaviours have been tested and validated in numerous previous studies against experimental recordings [7,34–36], we explored only three adjustable parameters of the regulatory action: the size of the affected BC cluster, the direction and the magnitude of the synaptic change.

### (b) Distributed versus volume-limited depression of excitatory input

**Figure 2.**
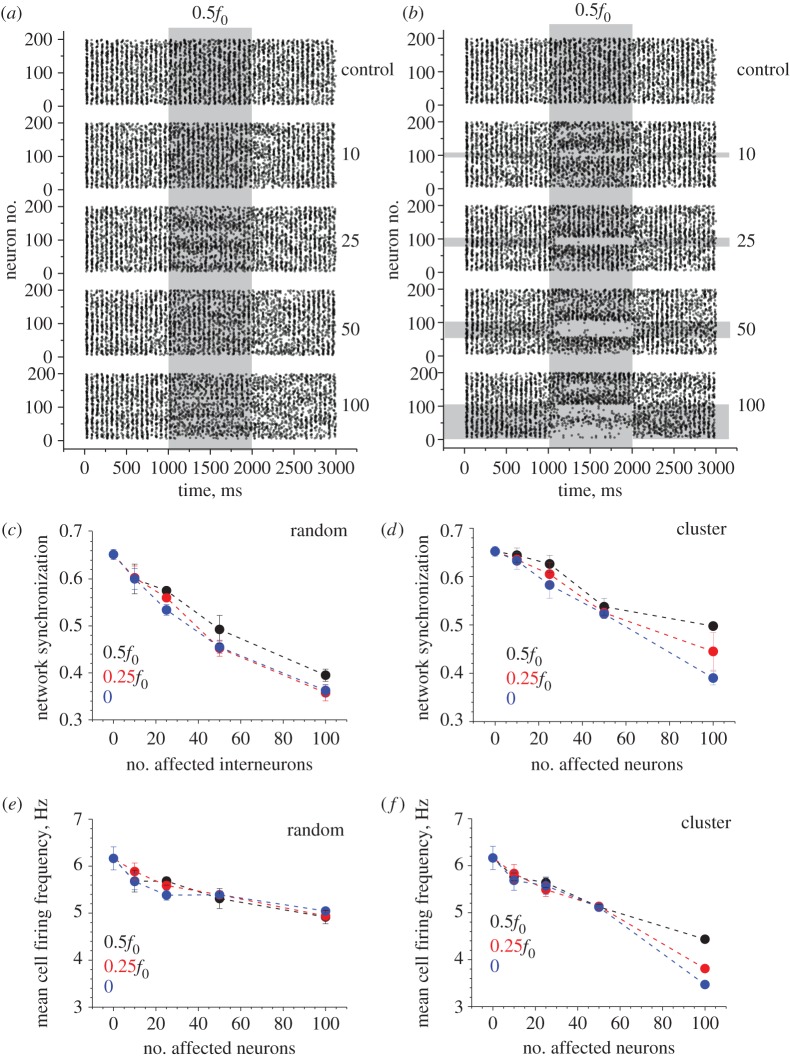
Depression of excitatory input in a proportion of basket cells reduces network synchronization and cell firing rates also depending on the spatial clustering of affected cells. (*a*) An example raster plot of cell firing (200 BCs) including the period of an inhibitory action. Numbers on the right (control, 10, 25, 50, 100) indicate the number of randomly selected BCs affected by the inhibitory action on their excitatory inputs. Shaded area labelled with 0.5*f*_0_ depicts halving of the baseline frequency *f*_0_ = 300 Hz (thus giving excitatory input at 150 Hz) over the designated 1000 ms period, as indicated. (*b*) A similar experimental design as in (*a*), but with the affected BCs grouped in spatial clusters of neighbours, as indicated by horizontal grey-shaded segments. Other notations as in (*a*). (*c*) The network synchronization parameter (§2d) measured throughout tests with a variable change in the excitatory input (0.5*f*_0_, 0.25*f*_0_ and 0 indicating silenced input) and variable numbers of affected BCs, as indicated. Data points show mean ± s.d. (*n* = 5 model runs throughout). (*d*) Results of an experiment similar to that shown in (*c*), but with the affected BCs grouped in spatial clusters of neighbours, as depicted in (*b*); other notations as in (*c*). (*e*) The mean cell firing rate (§2d) measured throughout tests with a variable change in the excitatory input (0.5*f*_0_, 0.25*f*_0_ and 0 indicating silenced input) and variable numbers of affected BCs, as indicated. Data points show mean ± s.d. (*f*) Results of an experiment similar to that shown in (*e*), but with the affected BCs grouped in spatial clusters of neighbours, as depicted in (*b*); other notations as in (*e*).

First, we simulated partial inhibition of excitatory inputs to BCs by reducing the average basal frequency *f*_0_ of the corresponding synaptic discharges (by 50%, 75% and by silencing altogether) over 1000 ms in a variable proportion (5–50%) of randomly selected networked BCs. (In this study, we did not consider a theoretically plausible feedback effect associating neuronal firing frequency with astroglial actions [48].) Second, to mimic the action of volume-limited astroglia, we constrained the above inhibitory action to the clusters of neighbouring BCs which contained the same numbers of individual cells, for comparison. Simulations showed that in both cases the inhibitory action had no appreciable effect on the oscillation frequency *Ω* (ranging from 18.1 ± 1.0 to 18.6 ± 0.5 Hz across the test runs compared with 18.0 ± 0.0 Hz in baseline conditions; mean ± s.d.; examples in figure 2*a,b*). At the same time, network synchronization was clearly reduced, with the greater effect seen in the case of randomly selected synapses (figure 2*c,d*). In contrast, the effect of inhibition on mean cell firing frequency *φ* was more pronounced in the case of the clustered (astroglia-like) inhibitory action (figure 2*e,f*). The raster plots of cell spiking indicated that space clustering of the effect also tended to produce highly uneven changes in cell firing patterns, with some cells becoming almost silent and others firing at a higher rate (figure 2*a,b*).

### (c) Distributed versus volume-limited facilitation of excitatory input

**Figure 3.**
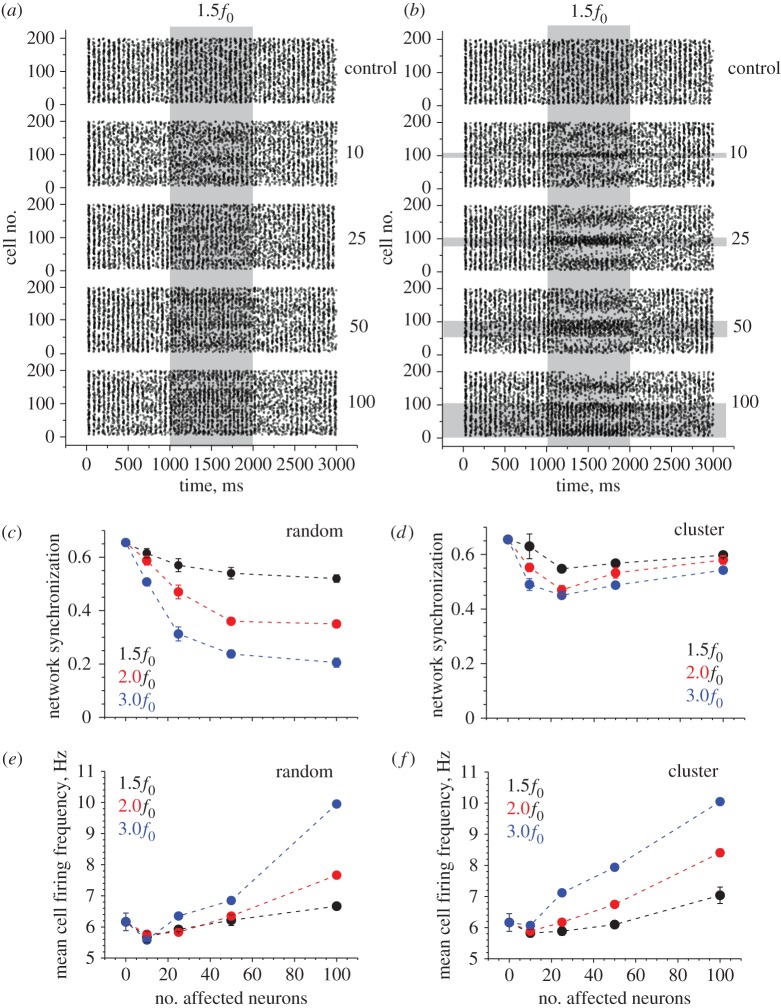
Facilitation of excitatory input in a proportion of basket cells alters network synchronization while increasing cell firing rates depending on the spatial clustering of affected cells. (*a*) An example raster plot of cell firing (200 BCs) including the period of an inhibitory action. Numbers on the right (control, 10, 25, 50, 100) indicate the number of randomly selected BCs affected by the inhibitory action on their excitatory inputs. Shaded area labelled with 1.5*f*_0_ depicts an increased frequency *f*_0_ = 300 Hz by 50% (thus giving excitatory input at 450 Hz) over the designated 1000 ms period, as indicated. (*b*) A similar experimental design as in (*a*), but with the affected BCs grouped in spatial clusters of neighbours, as indicated by horizontal grey-shaded segments. Other notations as in (*a*). (*c*) The network synchronization parameter (§2d) measured throughout tests with a variable change in the excitatory input (1.5*f*_0_, 2.0*f*_0_ and 3.0*f*_0_ indicating silenced input) and variable numbers of affected BCs, as indicated. Data points show mean ± s.d. (*d*) Results of an experiment similar to that shown in (*c*), but with the affected BCs grouped in spatial clusters of neighbours, as depicted in (*b*); other notations as in (*c*). (*e*) The mean cell firing rate (§2d) measured throughout tests with a variable change in the excitatory input (1.5*f*_0_, 2.0*f*_0_ and 3.0*f*_0_ indicating silenced input) and variable numbers of affected BCs, as indicated. Data points show mean ± s.d. (*f*) Results of an experiment similar to that shown in (*e*), but with the affected BCs grouped in spatial clusters of neighbours, as depicted in (*b*); other notations as in (*e*).

In line with the above approach (§3b), here we simulated facilitation of excitatory inputs to BCs by increasing the average basal frequency *f*_0_ of the corresponding synaptic discharges (by setting it at 150%, 200% or 300% of the baseline frequency *f*_0_ = 300 Hz), over 1000 ms in a variable proportion (5–50%) of randomly selected networked BCs. Again, to mimic the action of astroglia, we constrained the facilitatory action on the clusters of neighbouring BCs. Unlike the case of inhibition, these simulations revealed a small yet significant effect of synaptic facilitation on the network oscillation frequency *Ω* (ranging from 21.2 ± 2.4 to 21.2 ± 1.1 Hz across the runs compared with 18.0 ± 0.0 Hz in baseline conditions; mean ± s.d.; *p* < 0.009 at least across samples; examples in figure 3*a,b*). At the same time, network synchronization was prominently reduced in the case of randomly selected synapses, but not when using volume-limited cell clusters (figure 3*c,d*). Synaptic facilitation generally increased mean cell firing frequency *φ*, without much difference between the cases of distributed and clustered cells (figure 3*e,f*). However, the raster plots of cell spiking again indicated that volume-clustering, which is akin to astroglial effects, can produce highly uneven changes in cell firing patterns, with the range of firing frequencies evidently expanding (figure 3*a,b*). This is accompanied by what appears to be the development of a regular, or periodic, structure among subpopulations of firing cells, so that cell groups quasi-periodically occurring in space have similarly altered rates of firing (see lighter and darker areas raster plot areas during the facilitatory action in figure 3*a,b*).

## 4. Discussion

In this study, we focused on mechanisms that control oscillations in classical networks of interneurons and principal cells and thus play an important role in regulating rhythmic activities of the brain. The pattern of synaptic weights and their use-dependent changes is thought to underlie not only the oscillatory properties of neural networks, but also the fundamental principles of information transfer and memory formation in brain circuits. Recently, a growing body of experimental evidence has emerged pointing to a potentially important part played by astroglia in regulating the efficacy of local synapses. Because individual astrocytes occupy relatively restricted spatial domains in the synaptic neurophil, our aim here was to understand whether and how such volume-limited regulation of synaptic efficacy (which mimics astroglial actions) has a specific effect on network oscillations. To address this, we introduced space-constrained regulatory effects on synaptic inputs in a classical neural network model involving hippocampal interneurons (BCs) and principal neurons (pyramidal cells). Equipped with this approach, we compared the consequences of synaptic regulation, with or without its spatial clustering, while maintaining all other network parameters unchanged.

Our simulations have revealed several intriguing phenomena. First, we found it somewhat surprising that network oscillations appear to be quite resilient, especially in terms of the overall cell firing rate, to changes in the efficacy of excitatory drive (even synapse silencing) enacted in a significant proportion (up to 25%) of participating interneurons, be they clustered or selected randomly. In other words, the network seems to successfully redistribute the ‘firing load’ between cells that have been affected and unaffected by synaptic inhibition/facilitation, so that the overall numbers of generated spikes remain relatively unchanged. It would seem important to ascertain whether this phenomenon has any specific implications for preserving information coding in such networks.

Second, simulations have unveiled that when a large proportion of BCs (around 50%) are affected by a synaptic input change, space clustering of the regulatory influence (i.e. astroglia-like mode of action) has a substantially smaller impact on network synchronization compared with the evenly distributed action, be it inhibitory or facilitatory. Again, it would seem important to understand whether this suggests that the volume-limited effect of astroglia is ‘designed’ to preserve network oscillation properties while ensuring robust efficacy changes in local synaptic circuits. Finally, raster plots of cell firing throughout the tests suggest that during the regulatory action in the ‘astroglia mode’ there is some evident restructuring of interneuron population firing, both inside and beyond the affected cell cluster. Intriguingly, cell groups spaced at relatively regular intervals either increase or decrease their firing rates in a coherent fashion. This phenomenon suggests that volume-limited (astroglia-like) action could, at least in theory, prompt *spatial segmentation* of oscillating neural networks in terms of their firing behaviours. To understand an adaptive role of such phenomena in the information handling by neural circuits will require experimental techniques that would associate patterns of multiple cell firing with an established paradigm of memory trace formation in the network.
